# Tributyl phosphate as a type of environmental endocrine disruptor associated with liver fibrosis: insights from NHANES and *in vitro* validation

**DOI:** 10.3389/ftox.2025.1623830

**Published:** 2025-07-01

**Authors:** Liwei Yang, Aipeng Ju, Yawen Huang, Huibin Jiang, Jiaming Ye, Wen Qi, Liting Zhou

**Affiliations:** ^1^ Department of Occupational and Environmental Health, School of Public Health, Jilin University, Changchun, China; ^2^ Department of Human Anatomy, College of Basic Medical Sciences, Jilin University, Changchun, China

**Keywords:** EEDs, hepatic fibrosis, tributyl phosphate, NHANES, BRL-3A hepatocytes

## Abstract

**Background:**

Environmental endocrine disruptors (EEDs) had been proved as significant risk factors for liver fibrosis. However, which specific pollutants predominantly related to liver fibrosis remain unidentified. This study was aimed to screen in the specific EEDs using NHANES data and further validate the findings in BRL-3A hepatocytes.

**Methods:**

A total of 5,843 adult participants (≥18 years) incorporating data on EEDs/metabolites, demographics, lifestyle factors, and vibration-controlled transient elastography (VCTE) measurements were gated from the NHANES. Advanced analytical methods including LASSO regression, multivariable logistic regression, and restricted cubic spline (RCS) modeling were implemented for pollutant screening. *In vitro* validation involved treating BRL-3A hepatocytes with identified EEDs, followed by comprehensive assessment of fibrotic markers through quantitative PCR, Western blotting, and extracellular matrix component analysis.

**Results:**

Di-n-butyl phthalate (DBuP), the metabolites of tributyl phosphate (TBP), was demonstrated to be the strongest EEDs associated with liver fibrosis (*P* < 0.05). Mechanistic studies revealed that 1 μM TBP significantly elevated extracellular matrix components (HA: +130%, Ⅳ-Col: +22%) through MMP9 upregulation at both transcriptional (1.8-fold increase) and translational (1.73-fold increase) levels in hepatocytes.

**Conclusion:**

Our findings establish TBP as a novel environmental determinant positively correlated with liver fibrosis in U.S. adults. The profibrotic effects appear mediated through transcriptional activation of extracellular matrix remodeling genes, particularly via MMP9 pathway modulation.

## 1 Introduction

Liver fibrosis, characterized by the progressive accumulation of extracellular matrix (ECM) components, has exhibited a concerning upward trajectory in global incidence rates, emerging as a critical public health challenge. Recent epidemiological data reveal that advanced liver fibrosis affects approximately 3.3% of the general population (Zamani et al., 2024). This pathological process represents precursor to severe hepatic complications, including hepatocellular carcinoma (HCC) (Roehlen et al., 2020; Parola and Pinzani, 2019). Notably, emerging evidence implicates environmental endocrine disruptors (EEDs) as novel modifiable risk factors for fibrogenesis, with multiple cohort studies demonstrating significant exposure-disease associations (Qi et al., 2023; Quitete et al., 2024; Du et al., 2024).

EEDs, such as flame retardants (McGaha, 2024), metals (Yilmaz et al., 2020), organophosphate insecticides (Suwannarin et al., 2021), perfluoroalkyl and polyfluoroalkyl substances (Ding et al., 2020), as well as phthalates (Dalamaga et al., 2024), are widely used in food packaging materials, cosmetics, children’s toys and other daily necessities (de Paula and Alves, 2024; Li et al., 2024; Nicolopoulou-Stamati et al., 2015). EEDs predominantly enter human biological systems via three primary exposure pathways: dietary consumption, water intake, and dermal absorption from personal care products (Rashid et al., 2020; Gonsioroski et al., 2020). Epidemiological studies have identified diverse EEDs and their metabolic derivatives in human biofluids, with significant regional variations in concentration profiles.

Notably, median concentrations of mono-n-butyl phthalate (MBP) in general populations exhibited geographical disparities: Asian cohorts demonstrated levels of 13.4–147 ng/mL, compared to 9.30–57.6 ng/mL in American populations and 11.0–64.6 ng/mL in European demographics (Zhang et al., 2021). Recent biomonitoring data from Chengdu, China revealed peak tributyl phosphate (TBP) concentrations of 0.531 ng/mL in adult blood samples (Guo et al., 2023). Furthermore, urinary bis(1,3-dichloro-2-propyl) phosphate (BDCPP) levels among U.S. adults displayed substantial interindividual variability, ranging from 0.30 to 1.79 μg/day in population-based studies (Luo et al., 2020).

Upon exposure, EEDs circulate throughout the organism and undergo extensive metabolic processing followed by elimination. The liver plays a central role in this process, not only driving their metabolic transformation but also functioning as the key repository for their accumulation within the human body (Kabir et al., 2015). Many studies have identified a positive relationship between EEDs and liver fibrosis. An epidemiological study revealed that higher levels of heavy metals and polyfluoroalkyl substances (PFAS) in blood and urine samples are positively correlated with an increased risk for liver fibrosis (Wei et al., 2024). Additionally, animal research revealed that exposure to the organophosphorus pesticide chlorpyrifos in female rats results in their male offspring exhibiting a marked upregulation *Col1a1* mRNA expression at 8 weeks of age (Guibourdenche et al., 2021). Our previous research found that mono (2-ethylhexyl) phthalate (MEHP) promoted liver fibrosis by downregulating STAT5A in BRL-3A hepatocytes (Zhang et al., 2022). These findings suggest that EEDs may exacerbate liver fibrosis. However, most current research has primarily focused on the effects of single EEDs on the occurrence of liver fibrosis. In reality, humans are typically exposed to various kinds of EEDs. Therefore, identifying the specific pollutants within this mixture that predominantly associated with liver fibrosis, which could provide more targeted interventions for preventing liver fibrosis caused by EEDs exposure.

The National Health and Nutrition Examination Survey (NHANES) is a cross-sectional survey based on the health and nutritional status of adults and children in the United States. The NHANES provides detailed information on diseases and exposure levels to EEDs, which is applied to investigate the relationship between EEDs and liver damage (He et al., 2023; Li R. et al., 2022). In this paper, NHANES was utilized to identify the potential EEDs that related most significantly to the development of liver fibrosis. Furthermore, we investigated the effects of TBP, identified through the cross-sectional study, on BRL-3A hepatocytes by measuring the levels of ECM components and matrix metalloproteinases 2 (MMP-2) and 9 (MMP-9) mRNA and protein. Our findings provide a more valuable target for the prevention of liver diseases caused by EEDs.

## 2 Methods

### 2.1 Screening the major EEDs promoting liver fibrosis based on NHANES

#### 2.1.1 Data source and study population

The NHANES is a nationally representative cross-sectional survey that enrolls participants using a stratified, multistage, clustered probability sampling frame. This design ensures representativeness of the civilian, non-institutionalized U.S. population. It is conducted by the National Center for Health Statistics (NCHS), which is a part of the Centers for Disease Control and Prevention. The protocols of NHANES are approved by the NCHS institutional review board. Written informed consent is obtained from all participants. This paper analyzes data from 2017-2018 cycle years. The data from this analysis are publicly available at https://wwwn.cdc.gov/nchs/nhanes/Default.aspx.

**FIGURE 1:**
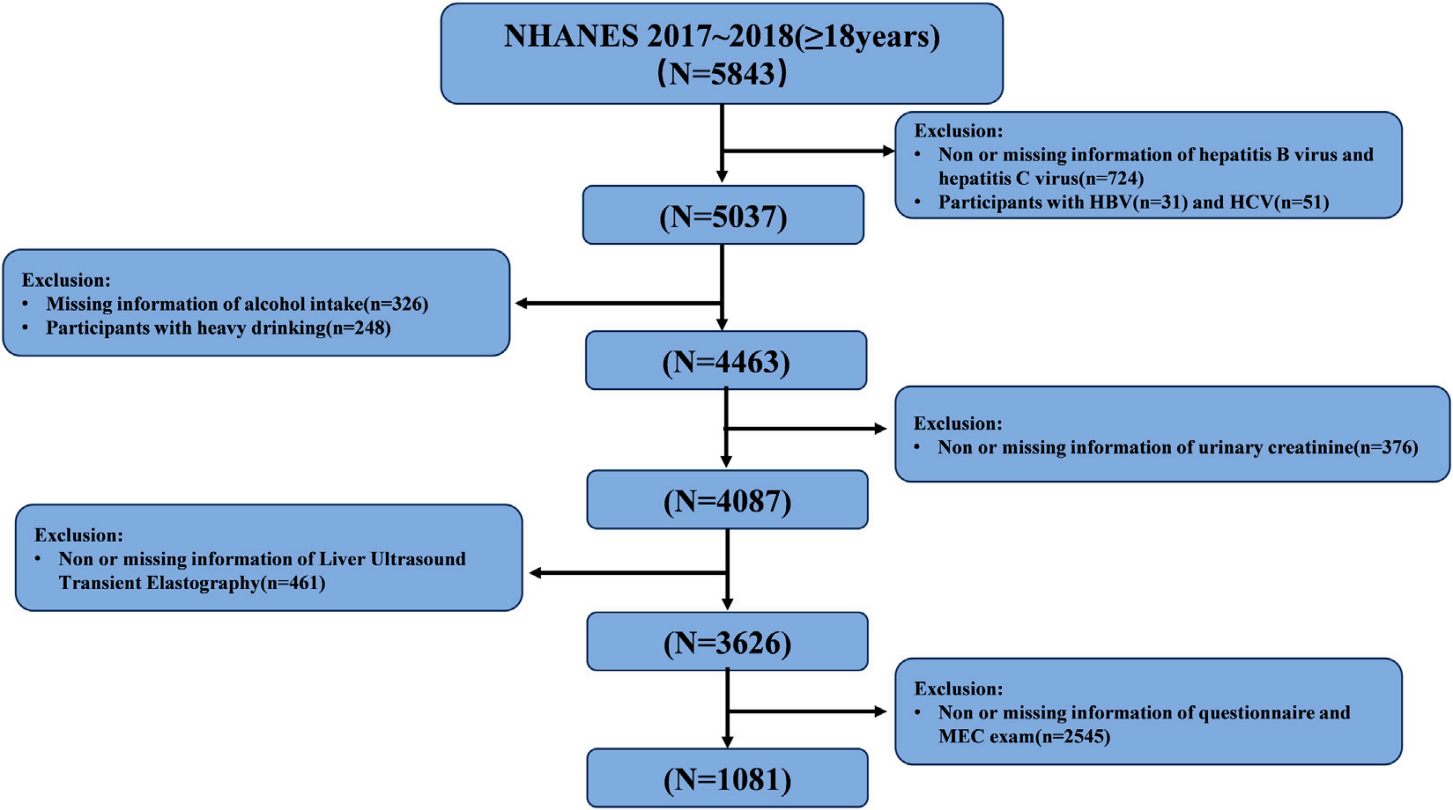
Flow-chart of the study participants selected from National Health and Nutrition Examination Survey (NHANES) 2017–2018.

A total of 5,843 participants aged 18 years or older (with a mean aged of 49 years) from the U.S. were included in our research. All participants had both EEDs and their metabolites levels measured, along with VCET data. The exclusion criteria included (Zamani et al., 2024): Infected with hepatitis B or hepatitis C (Roehlen et al., 2020); Heavy alcohol consumption, defined as more than four drinks on any day or more than 14 drinks/week for men, and more than three drinks on any day or more than seven drinks/week for women (Warner et al., 2022); (Parola and Pinzani, 2019) Abnormal urine creatinine level (<20 mg/dl or >300 mg/dl) (Merida-Ortega et al., 2022); (Qi et al., 2023) Invalid liver ultrasound transient elastography data (Quitete et al., 2024); Missing data of covariant. Ultimately, this study included 545 male participants and 536 female participants. The detailed inclusion and exclusion process is shown in Figure 1.

#### 2.1.2 Detection method and definition of liver fibrosis

VCTE is a tool used to measure liver stiffness (LS) using the Fibroscan device (Echosens, Paris, France) which can estimate fibrosis (Mikolasevic et al., 2016). Invalid data were excluded if participants met any of the following criteria (Zamani et al., 2024): Inability to lie down on the exam table (Roehlen et al., 2020); Pregnant (Parola and Pinzani, 2019); Presence of an implanted electronic medical device, a bandage, or lesions near the right ribcage area. All eligible participants were required to fast for at least 3 h prior to examination and received at least 10 complete stiffness measurements using two types of transducers: medium (M, 3.5 MHz) or extra-large (XL, 2.5 MHz). Valid data were considered when the interquartile range (IQR) of liver stiffness did not exceed 30% of the median stiffness value. According to previous studies, liver stiffness values ≥ 8 kPa were defined as fibrosis (Wan et al., 2022). Furthermore, participants with cirrhosis, defined by liver stiffness values ≥13 kPa, were not excluded from the study, as fibrotic lesions can also coexist in the context of cirrhosis (Gines et al., 2021).

#### 2.1.3 EEDs selected in the research

This paper investigated five types of EEDs that are associated with liver fibrosis: flame retardants, metals, organophosphate insecticides, perfluoroalkyl and polyfluoroalkyl, phthalates and plasticizers and their corresponding metabolites. While most of these substances have established links to hepatotoxicity, their involvement in liver fibrosis remains uncertain. The Supplementary Tables S1, S2 provide the specific EEDs and their detection methods.

#### 2.1.4 Covariates

The variables associated with liver fibrosis or the levels of EEDs and their metabolites, including sex, age, race, educational level, smoking status (Nigra et al., 2021), drinking status (Warner et al., 2022), Body Mass index (BMI), activity status, blood pressure, and cholesterol level were selected as covariate. The specific classification of covariates and its basis were showed in Table 1.

**TABLE 1:** Stratification of covariates.

Covariates	Stratification	Basis
Age		
	<40	
	40–59	
	>59	
Race		
	Mexican American	
	Other Hispanic	
	Non-Hispanic White	
	Non-Hispanic Black	
	Other races	
Educational level		
	Below high school	
	High school	
	Above high school	
Smoking status (Nigra et al., 2021)		
	Never	Smoked less than 100 cigarettes in their lifetime
	Former	Smoked more than 100 cigarettes in their lifetime but currently did not smoke
	Current	Smoked more than 100 cigarettes in their lifetime and currently smoked
Drinking status (Warner et al., 2022)		
	Never drinking	Never consumed at least one alcoholic drink in their lifetime
	No-drinking	Consumed alcoholic drinks in the past but did not drink in the last 12 months
	Moderate drinking	Man who reported an average consumption of 2 drinks or less per day, and women who reported an average consumption of 1 drink or less per day
BMI		
	Normal	<25 kg/m^2^
	Overweight	≥25 and <30 kg/m^2^
	Obese	≥30 kg/m^2^
Physical activity		
	Strong physical activity	
	Moderate physical activity	
	No physical activity	
Hypertension		
	Yes	systolic blood pressure ≥140 mmHg, and diastolic blood pressure ≥90 mmHg, or self-reported history of hypertension diagnosed by physicians
	No	

#### 2.1.5 Data pre-processin

All EEDs metabolites measured in urine samples were corrected for creatinine levels. Spearman correlation analysis was used to explore the correlation between variables, and the results are presented in Supplementary Figure S1. Variables with Spearman coefficients greater than 0.8 are listed in Supplementary Table S3. Notably, no variables showed correlations above 0.95, which is considered indicative of high collinearity. Therefore, all variables were retained for further analysis.

Prior to proceeding with subsequent analyses, box plots were generated for all variables to identify the outlier values which were defined as beyond the box boundaries (1.5 times the interquartile range). We deleted the outliers and used multiple imputation to imputation the missing value through the R package “mice”. Supplementary Table S4 shows the number of missing values of EEDs. Supplementary Figure S2 shows the distribution of fill values and original data. It could be found the distribution of the two types of data are approximately the same.

#### 2.1.6 Baseline data analysis

The participants were categorized into two groups: those with liver fibrosis and those without liver fibrosis based on the baseline data. Participant characteristics were presented using medians for continuous variables and percentages for categorical variables. The EEDs levels were presented as the median (95% confidence intervals). Variables Screen and regressive analysis.

Firstly, LASSO was used for determining the penalized model coefficients and perform variable selection. A 3-fold cross-validation was employed to identify the model with the lowest mean square error (MSE), which was used to determine the best LASSO model. The importance of variables on liver fibrosis were reflected by the absolute value of the compressed model coefficients.

Secondly, logistic regression was conducted to evaluate the strength and direction of associations between the selected EEDs and liver fibrosis. The EEDs were categorized into tertiles based on their concentration levels. Odds ratios (ORs) and 95% CIs for liver fibrosis were calculated for the second and third tertiles, using the first tertile as the reference category. Four distinct logistic models were established as follows (Zamani et al., 2024): Univariate model without covariates (Roehlen et al., 2020); Univariate model with covariates (Parola and Pinzani, 2019); Multivariate model without covariates (Qi et al., 2023); Multivariate model with covariates.

Finally, to enhance the accuracy of this research, Restricted Cubic Splines (RCS) were performed to examine both linear and non-linear dose-response relationships between EED concentrations and liver fibrosis outcomes.

### 2.2 The effects of TBP on the liver fibrosis in BRL-3A hepatocyte

#### 2.2.1 Cell culture, differentiation and treatment

BRL-3A hepatocytes were gated from the Cell Bank, Chinese Academy of Science. The cells were cultured in DMEM medium (Gibco, United States) supplemented 10% fetal bovine serum (BI, Kibbutz Beit Haemek, Israel) at 37°C and 5% CO_2_. BRL-3A hepatocytes were seeded into 6-well plates (2 × 10^6^ cells/well) and treated with different concentrations of Tributyl phosphate (TBP) (10^–5^, 10^–6^ and 10^–7^ M) for 24 h. The lowest concentration set in this study was comparable to environmental monitoring levels (10^–7^ mol/L was equivalent to 26.6–43.1 ng/mL of the tested TBP) (Zhao et al., 2016; Li et al., 2017). The control group was treated with equal volume of DMEM, while 1%DMSO was set as solvent control.

#### 2.2.2 Cell viability assessment

CCK-8 assay kit (Dojindo, Japan) was used to detect the viability of BRL-3A cells. The cells were seeded into 96-well plates at a density of 1 × 10^5^ cells/well and subsequently exposed to TBP at concentrations of 10^–7^, 10^–6^ and 10^–5^ M for 24 h. Two hours before the ending of exposure period, the absorbance of each well was measured using a microplate reader at 450 nm. The survival rate of the BRL-3A cells was calculated according to the absorbance values.

$$Cell\;survival\;rate\left(\%\right)=\frac{\left(Sample\;hole\;A450–Culture\;solution\;hole\;A450\right)}{\left(Control\;hole\;A450–Culture\;solution\;A450\right)}x100\%$$

#### 2.2.3 Extracellular matrix detection

The levels of Collagen Type Ⅳ(Ⅳ-Col) and hyaluronic acid (HA) secreted by BRL-3A hepatocytes were detected with ELISA commercial kits (Shanghai Langdun Bioengineering Institute, China). The detection limits of Ⅳ-Col and HA were 1.5–240 ng/mL and 0.15–24 ng/mL respectively and the CVs of Ⅳ-Col and HA were all<12%.

#### 2.2.4 RNA extraction and real time-PCR analysis

The total RNA was extracted using Trizol reagent (Invitrogen, United States). 500 ng RNA was reverse transcribed into cDNA using Strand cDNA Synthesis Kit (Tolobio, China). Real-time PCR was performed with a SYBR Green Real-time PCR kit (Tolobio, China). Supplementary Table S5 shows the primer sequences for *MMP2* and *MMP9* used for amplification of each gene, and *GAPDH* was used as an internal reference gene.

#### 2.2.5 Western blot

The total cellular protein was extracted with RIPA Lysis Buffer (Solarbio, China). The concentration of protein was measured by BCA protein assay kit (Beyotime, China). Equal amounts of protein (20 μg protein/well) were loaded onto an SDS-PAGE gel and then transferred to a nitro-cellulose membrane. The membranes were blocked with 5% nonfat milk in PBS-Tween-20 for 2 hours and then were incubated with anti-rabbit MMP2 (Proteintech, United States), anti-rabbit MMP9 (Proteintech, United States) and anti-mouse GAPDH (Proteintech, United States) overnight at 4°C. The specificity of antibodies were shown in the Supplementary Figure S3. After washing with Tris-buffered saline and Tween 20 three times, secondary antibodies that could specifically bind to the primary antibody were added. An enhanced chemiluminescence kit (Proteintech, United States) was used to visualize the protein bands, and the gray values of the bands were analyzed by Image-Pro Plus 6.0 software.

#### 2.2.6 Statistical analysis

All data were analyzed by R4.0.2 and IBM SPSS 24.0, and each experiment was performed independently at least three times. The differences among different groups were compared by One-way ANOVA or Rank Sum Test with the LSD or Kruskal–Wallis test between two groups. A two-tailed *P* value <0.05 was considered statistically significant.

## 3 Results

### 3.1 Screening the primary EEDs promoting liver fibrosis based on NHANES

#### 3.1.1 Demographic characteristics

Table 2 presents the demographic characteristics of participants divided into those with liver fibrosis and those without. Compared to participants without liver fibrosis, those with liver fibrosis exhibited higher frequencies of older age, smoking behavior, physical inactivity, obesity, and hypertension (*P* < 0.05).

**TABLE 2:** The characters of participants.

Characteristic	Total	Fibrosis	Non- fibrosis	*P* ^a^
N	1,081	95	986	
Sex(n,%)				
Female	536 (49.6)	46 (48.4)	490 (49.7)	0.897
Male	545 (50.4)	49 (51.6)	496 (50.3)	
Age(n,%)				
mean	49 (18.3)	56 (16.0)	49 (18.4)	<0.001
18–39	364 (33.7)	16 (16.8)	348 (35.3)	<0.001
40–59	330 (30.5)	31 (32.6)	299 (30.3)	
≥60	387 (35.8)	48 (50.5)	339 (34.4)	
Race-Ethnicity(n,%)				
Mexican American	166 (15.4)	18 (18.9)	148 (15.0)	0.314
Non-Hispanic Black	221 (20.4)	22 (23.2)	199 (20.2)	
Non-Hispanic White	387 (35.8)	32 (33.7)	355 (36.0)	
Other Hispanic	108 (10.0)	12 (12.6)	96 (9.7)	
Other Race	199 (18.4)	11 (11.6)	188 (19.1)	
Eduction (n,%)				
Below high school	201 (18.6)	20 (21.1)	181 (18.4)	0.634
High school	273 (25.3)	26 (27.4)	247 (25.1)	
Above high school	607 (56.2)	49 (51.6)	558 (56.6)	
Smoke (n,%)				
Current	156 (14.4)	13 (13.7)	143 (14.5)	<0.001
Ever	249 (23.0)	37 (38.9)	212 (21.5)	
Never	676 (62.5)	45 (47.4)	631 (64.0)	
Alcohol(n,%)				
Non-drinker	222 (20.5)	32 (33.7)	190 (19.3)	0.004
Never drinker	112 (10.4)	9 (9.5)	103 (10.4)	
Moderate drinker	747 (69.1)	54 (56.8)	693 (70.3)	
Active (n,%)				
Moderate active	242 (22.4)	21 (22.1)	221 (22.4)	<0.001
Non-active	546 (50.5)	64 (67.4)	482 (48.9)	
Vigorous active	293 (27.1)	10 (10.5)	283 (28.7)	
BMI(n,%)				
Normal	301 (27.8)	8 (8.4)	293 (29.7)	<0.001
Obese	437 (40.4)	71 (74.7)	366 (37.1)	
Over weight	343 (31.7)	16 (16.8)	327 (33.2)	
Blood pressure (n,%)				
Hypertension	458 (42.4)	56 (58.9)	402 (40.8)	0.001
Non-hypertension	623 (57.6)	39 (41.1)	584 (59.2)	
Total Cholesterol(mg/dL)	4.76 (4.11, 5.51)	4.76 (3.95, 5.51)	4.7 (4.11, 5.51)	0.404
Urinary Creatinine(g/dL)	1.16 (0.66, 1.68)	1.27 (0.76, 1.82)	1.15 (0.64, 1.66)	0.157

Data are presented as the median (Inter Quartile Range) or frequency (percentage).

^a^ Rank sum test was performed for continuous variables, and Chi-square test was performed for categorical variables.

#### 3.1.2 The characteristics of EEDs

Supplementary Table S6 presents the levels of EEDs and their metabolites in human urine or blood, categorized according to the presence or absence of liver fibrosis. Notably, participants with liver fibrosis exhibited significantly lower concentrations of Dibutyl phosphate (DBuP), Di-ethylthiophosphate (DETP), Dimethyldithiophosphate (DMDP), Perfluorooctane sulfonamide (PFDeA), Mono-2-methyl-2-hydroxypropyl phthalate (MHiBP), and Mono-3-hydroxy-butyl phthalate (MHBP). Conversely, higher levels of Mono(2-ethyl-5-carboxypentyl) phthalate (MECPP) and Mono-oxo-isononyl phthalate (MONP) were observed in this group (*P* < 0.05).

#### 3.1.3 Screening of EEDs associated with liver fibrosis

**FIGURE 2:**
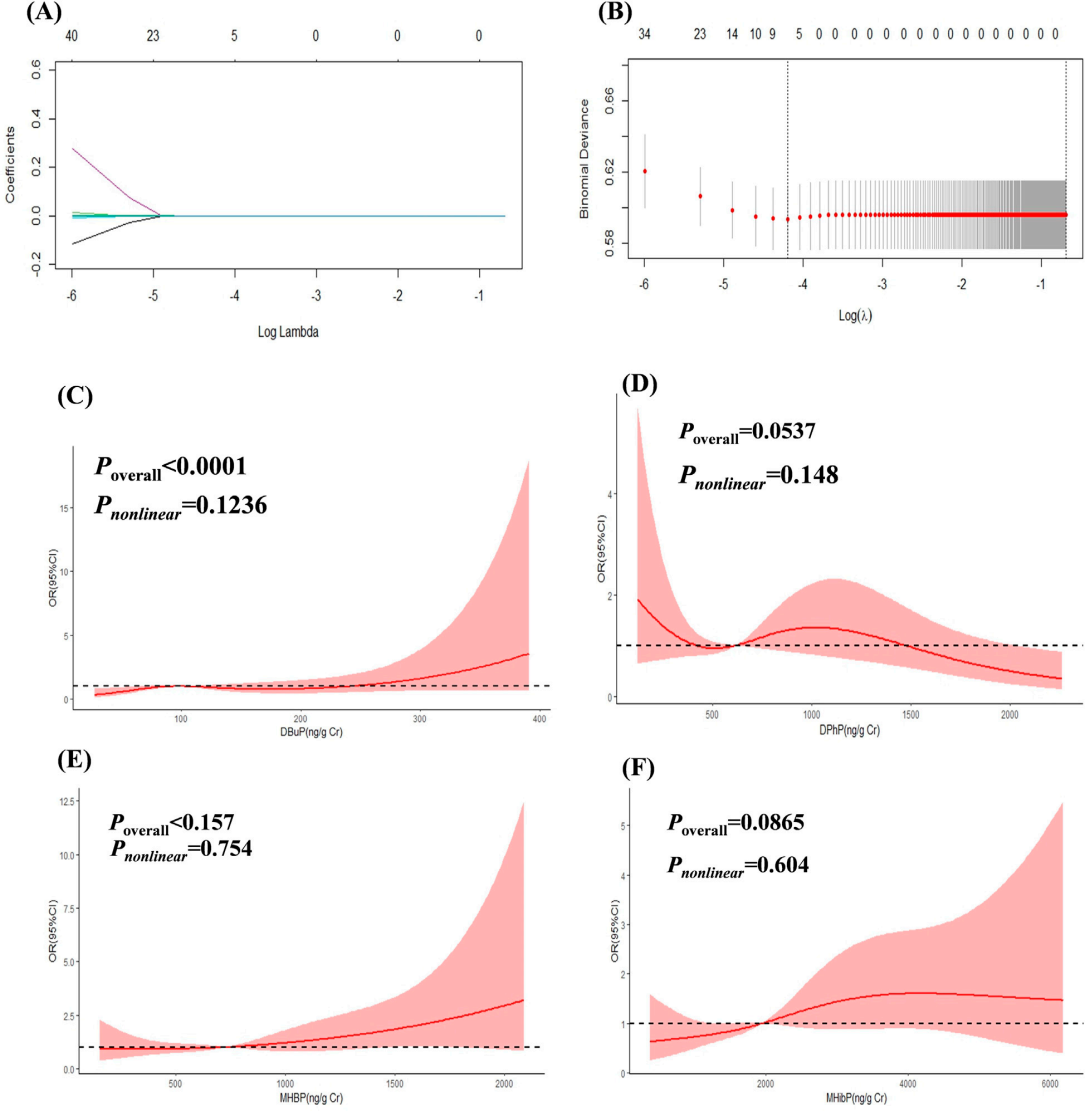
The Association between EEDs and liver fibrosis. **(A)** showed the change of prediction error of LASSO regression model with penalty parameter log λ of liver fibrosis. **(B)** showed the filtering path of the LASSO regression models of liver fibrosis. Covariates were not displayed in the picture in order to understanding better. **(C)** RCS curves of DBuP (ng/Cr) and liver fibrosis. **(D)** RCS curves of DPhP(ng/Cr) and liver fibrosis. **(E)** RCS curves of MHBP(ng/Cr) and liver fibrosis. **(F)** RCS curves of MHibP (ng/Cr) and liver fibrosis.

The results of LASSO analysis demonstrated that DBuP, MHBP, MHibP and Diphenyl phosphate (DPhP) exhibited significant associations with liver fibrosis (Figures 2A,B). The coefficients of the variables after compression in the model by LASSO were provided in regression analysis. Among these, DBuP, as the TBP metabolites, exhibited the highest coefficient in the models (Table 3).

**TABLE 3:** The coefficients of variable in the model by LASSO.

Variables	Beta coefficients
DBuP	4.812,702 × 10^−4^
MHBP	3.488405 × 10^−5^
MHibP	2.893609 × 10^−5^
DPhP	−3.873801 × 10^−6^

#### 3.1.4 The associations between EEDs with liver fibrosis

The concentration of DBuP had a positively association with liver fibrosis in the fully adjusted models (*P* < 0.05) in Logistic regression analysis (Table 4). The highest concentrations of MHBP and MHiBP showed a positively association with liver fibrosis in the univariate model overal (*P* < 0.05).

**TABLE 4:** Association between EEDs and liver fibrosis using logistic regression.

Variables	Model 1	Model 2	Model 3	Model 4
DBuP				
Quartile 1	1.00	1.00	1.00	1.00
Quartile 2	1.69 (1.04–2.72)^*^	1.85 (1.09–3.12)^*^	1.61 (0.99–2.60)	1.79 (1.05–3.05)^*^
Quartile 3	1.89 (1.06–3.46)^*^	2.30 (1.21–4.47)^*^	1.64 (0.9–3.04)	2.08 (1.08–4.10)^*^
DPhP				
Quartile 1	1.00	1.00	1.00	1.00
Quartile 2	0.88 (0.50–1.48)	0.92 (0.50–1.62)	0.8 (0.46–1.36)	0.81 (0.44–1.45)
Quartile 3	0.76 (0.41–1.38)	0.67 (0.34–1.28)	0.62 (0.33–1.13)	0.53 (0.26–1.05)^*^
MHBP				
Quartile 1	1.00	1.00	1.00	1.00
Quartile 2	1.15 (0.70–1.85)	1.13 (0.66–1.88)	1.04 (0.62–1.72)	0.95 (0.54–1.65)
Quartile 3	2.04 (1.08–3.99)^*^	1.99 (1.01–4.08)^*^	2.06 (0.99–4.47)	1.32 (0.6–2.95)
MHibP				
Quartile 1	1.00	1.00	1.00	1.00
Quartile 2	1.11 (0.68–1.79)	1.18 (0.69–1.97)	1.02 (0.61–1.70)	1.09 (0.62–1.90)
Quartile 3	2.39 (1.24–4.85)^*^	2.60 (1.29–5.51)^*^	1.5 (0.73–3.15)	2.3 (1.04–5.30)

Data were presented as the OR (95%CI). Model 1: Variables were included individually without adjusting for covariates. Model 2: Variables were included individually adjusting for age, year, race, education, smoking, BMI, blood pressure; TC, active status and drinking. Model 3: Variables were included mixed without adjusting for covariates. Model 4: Variables were included mixed adjusting for age, year, race, education, smoking, BMI, blood pressure; TC, active status and drinking. *P < 0.05.

The linear and dose-response relationships between EEDs and liver fibrosis were shown in Figures 2C–F. DBuP, MHBP, MHibP and DPhP all exhibited a linear relationship with liver fibrosis. The value of OR of liver fibrosis significantly increased with the enhancing concentration of DBuP (*P* < 0.05).

### 3.2 Effects of TBP on the liver fibrosis

#### 3.2.1 The effect of TBP on cell survival rate

Supplementary Figure S4 showed the cell survival rate of BRL-3A. With the increased levels of TBP, the cell survival rate showed a trend of first increasing and then decreasing. The TBP dose selected in this study did not induce significant cytotoxic effects on cells and is suitable for subsequent research.

#### 3.2.2 The effect of TBP on the extracellular matrix levels in BRL-3A hepatocytes

**FIGURE 3:**
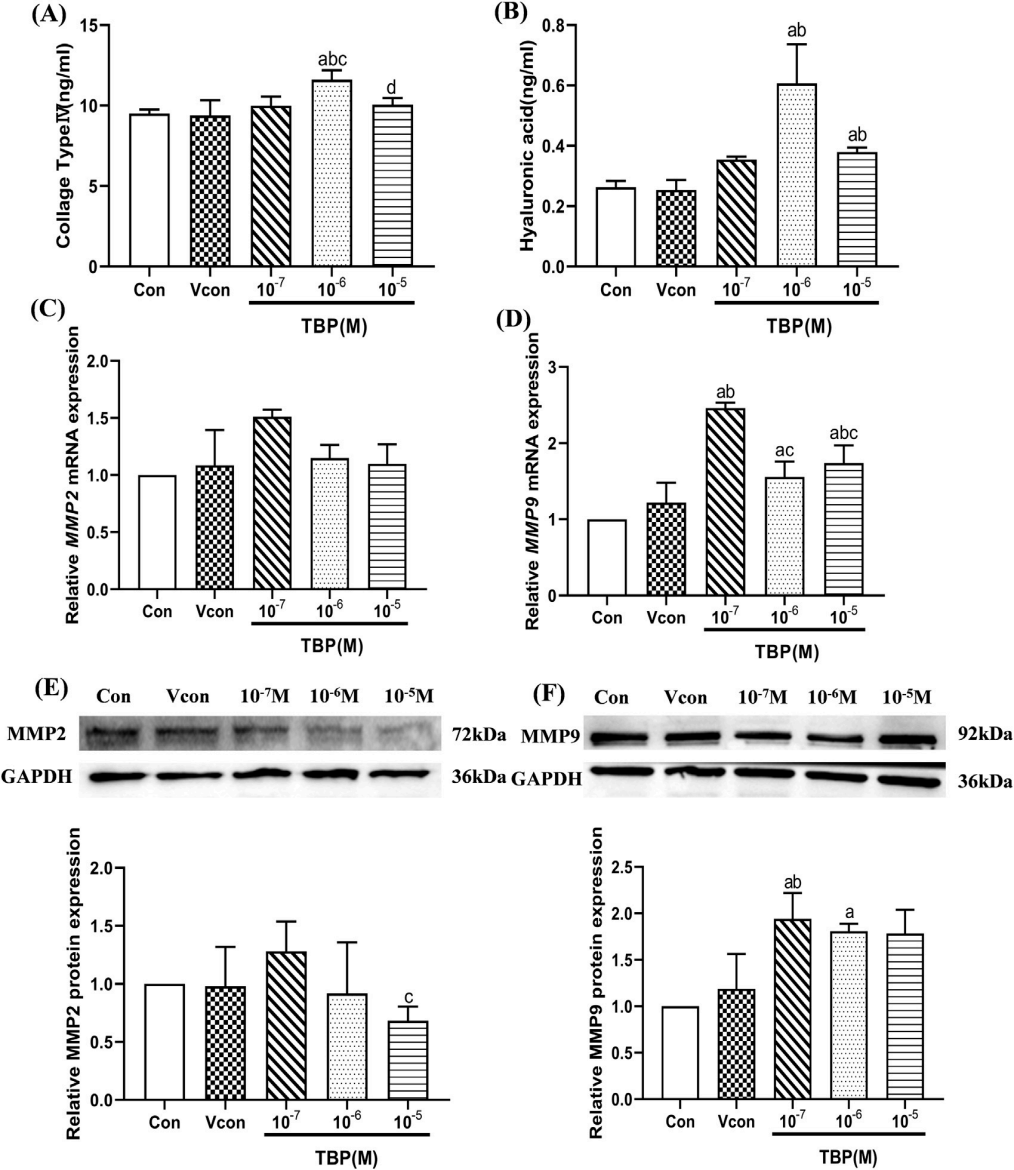
The levels of extracellular matrix and fibrosis genes in BRL-3A hepatocytes. **(A)** The levels of IV Col (ng/mL) in BRL-3A hepatocytes; **(B)** The levels of HA (ng/mL) in BRL-3A hepatocytes; **(C)** The relative mRNA expression levels of *MMP2* in BRL-3A hepatocytes; **(D)** The relative mRNA expression levels of *MMP9* in BRL-3A hepatocytes; **(E)** The relative protein expression levels of MMP2 in BRL-3Ahepatocytes; **(F)** The relative protein expression levels of MMP9 in BRL-3A hepatocytes; ^a^Comparison with 0 M TBP (*P* < 0.05); ^b^Comparison with solvent control (*P* < 0.05); ^c^Comparison with 10^–7^ M TBP (*P* < 0.05); ^d^Comparison with 10^–6^ M TBP (*P* < 0.05); Values are means ± SD (n ≥ 3).

Given that DBuP demonstrated a significant association with liver fibrosis in mixed EEDs within the NHANES dataset, we further investigated the harmful effect and mechanisms of TBP on the liver fibrosis. Two key representative components of the extracellular matrix, hyaluronic acid (HA) and type IV collagen (Ⅳ-Col), were quantified as surrogate markers for assessing liver fibrosis. Compared with the control group, the secretion levels of HA in BRL-3A hepatocytes were increased by 1.44-flod and 2.30-fold at concentrations of 10^–5^ and 10^–6^ M TBP, respectively (*P* < 0.05). Similarly, the levels of type IV collagen (Ⅳ-Col) were also elevated by 1.05-flod and 1.66-flod under the same conditions (Figures 3A,B).

#### 3.2.3 Effects of TBP on the mRNA and protein levels of MMP2 and MMP9 in BRL-3A hepatocytes

The expression levels of MMP2 and MMP9 mRNA and protein in BRL-3A hepatocytes are shown in Figures 3C–G. Compared with the control group, the expression level of MMP9 mRNA and protein was obviously increased by 1.94-flod and 1.80-flod at the 10^–7^ and 10^–6^ M TBP groups, respectively (*P* < 0.05). However, TBP did not significantly promote the expression of MMP2.

## 4 Discussion

Exposure to EEDs has been linked to hepatic toxicity. However, limited research has been conducted to elucidate the specific compounds within these EEDs that are primarily related to the progression of liver fibrosis. In this study, we employed a combination of LASSO, logistic and RCS to identify the specific substance showing the strongest association with liver fibrosis among the mixtures EEDs analyzed from NHANES data. To further investigate its effects and underlying mechanisms, BRL-3A hepatocytes were treated with TBP, a compound identified through our statistical models. This study provides a target for preventing liver fibrosis caused by EEDs.

The LASSO is a statistical technique for variable selection and regularization, which has been extensively employed in studies examining the associations between environmental pollutants and their adverse health effects. For instance, LASSO was used to identify organic pollutants associated with disrupted female hormone levels (Peng et al., 2023). Furthermore, Amine (Amine et al., 2023) reported that prenatal exposure to methylparaben is a significant predictor of lower general health scores during early life and childhood. In this study, we first identified the EEDs associated with liver fibrosis by utilizing the LASSO approach. The results indicated that DBuP, MHBP, MHiBP and DPhP all exhibited significant positive correlations with liver fibrosis, with DBuP notably demonstrating the strongest correlation among these variables. The logistic regression analysis indicated that higher concentrations of DBuP are positively associated with an increased risk of liver fibrosis. However, this study observed lower urinary levels of DBuP in individuals with liver fibrosis. Previous research has demonstrated that liver damage, such as fibrosis, can impact drug metabolism by altering the expression of metabolic enzymes and transferrin (Palatini and De Martin, 2016; Congiu et al., 2009). Numerous environmental pollutants can impair the liver’s metabolic function (Zh et al., 2024; Maerten et al., 2024). Moreover, research has demonstrated that the accumulation of TBP in the liver is significantly higher compared to that in other tissues and organs (Choo et al., 2018; Bekele et al., 2018). Based on these theory and results, we hypothesized that liver fibrosis may impair the metabolic capability of chemicals to which individuals are exposed, which could, in turn, result in reduced levels of their metabolites. The results of cross - sectional studies suggest that DBuP, MHBP, MHiBP, and DPhP may be associated with liver fibrosis to a certain extent. Among these substances, DBuP appears to have the most pronounced correlation. Moreover, the fibrogenic effects of MHBP (Huo et al., 2023), MHiBP (Jin et al., 2023), and DPhP (Chu and Letcher, 2019) have been previously reported. Consequently, in our *in vitro* studies, we will focus on investigating the impact of TBP on the progression of liver fibrosis.

A key methodological limitation of cross-sectional studies is their inherent inability to definitively establish a temporal causal relationship between TBP exposure and the development of liver fibrosis. Hence, we assessed the expression levels of extracellular matrix-related and fibrosis-associated genes in BRL-3A hepatocytes. Existing research has demonstrated that changes in the secretion levels of IV Col and HA, as well as the gene expression of MMP2 and MMP9 in BRL-3A cells, can reflect liver fibrosis in the body to a certain extent. In this study, TBP treatment significantly elevated the levels of IV Col and HA. Furthermore, both the mRNA and protein levels of MMP9 were markedly upregulated in the TBP - treated group. Despite the data from RCS and BRL-3A hepatocyte studies, no clear dose–response relationship was observed between TBP exposure and liver fibrosis. A study found 1 µM TBP induced a greater increase in total triglyceride levels compared to 10 µM TBP in HepG2 cells (Hao et al., 2019). Our previous research identified a non - monotonic dose - response relationship between MEHP and the Noth signaling pathway in 3T3-L1 cells. EEDs have a characteristic of nonmonotonic dose–response (Wu et al., 2024). We hypothesize that the nonlinear relationship stemming from TBP might be associated with the threshold effect of EEDs on bodily harm. However, additional research is imperative to validate this hypothesis.

DBuP, the primary metabolite of TBP, serves as a flame retardant, defoamer and rubber plasticizer in various applications within daily life (Garcia et al., 2007). Global consumption of TBP has reached an annual volume ranging between 3,000 and 5,000 metric tons, with a sustained compound annual growth rate (CAGR) of 4%–5% observed in recent years (Ahire et al., 2012). TBP has been detected in various environmental media, including indoor air, surface water, atmosphere, dust, sediments, and soil (Li S. et al., 2022; Zhang et al., 2020). Humans are exposed to TBP through both diet and airborne pathways, with its metabolites having been detected in human urine samples in various studies (Yoshida et al., 2022; Fromme et al., 2014). Extensive studies have consistently demonstrated the hepatotoxic effects of TBP. Research has shown that DBuP is linked to liver function impairment in adolescent populations (Li R. et al., 2022). Additionally, in the adult population in the United States, increased concentrations of urinary DBuP have been associated with a higher risk of nonalcoholic fatty liver disease (NAFLD) (Chai et al., 2022). Animal studies revealed that C57BL/6 mice exposed to 30 mg/kg TBP for 14 days exhibited significant increases in binuclear hepatocytes and nuclear hyperchromatic cells in liver pathological sections (Zhou et al., 2017). Furthermore, Elvira Mennillo’s research (Mennillo et al., 2019) demonstrated that oxidative stress enzymes, including glutathione S-transferase (GSTs), which play a key role in the conjugation of xenobiotics, were elevated in a rat hepatic cell-line (H4IIE) exposed to 50 μM and 100 µM TBP. Our study represents the initial discovery of the association between TBP and liver fibrosis in the human population. This finding further corroborates the hepatotoxic effects of TBP.

However, there are some limitations to this research. First, the investigation was restricted to five specific EEDs available in the NHANES dataset, potentially overlooking other environmentally relevant endocrine disruptors and limiting the comprehensive assessment of chemical exposures. Second, while our *in vitro* cellular models provided initial insights into TBP-induced hepatic fibrosis mechanisms, the use of BRL-3A hepatocytes has limitations when extrapolating to human disease. In the future, we should utilize models like human liver organoids to better simulate the complex structure and function of the human liver, thereby enabling a more accurate evaluation of the impact of TBP on liver fibrosis. Finally, our analysis did not account for potential synergistic interactions with other biological and environmental factors, including concomitant medication use, dietary components, and co-exposure to unmeasured environmental contaminants, which could collectively influence hepatic outcomes.

## 5 Conclusion

TBP was filtered from the mixture of EEDs that are associated with liver fibrosis. After treatment with TBP, the BRL-3A hepatocytes exhibited increased secretion levels of extracellular matrix and fibrosis-related genes. Our study provided an accurate target for the prevention of the damaging effects of EEDs on liver fibrosis.

## Data Availability

The original contributions presented in the study are included in the article/Supplementary Material, further inquiries can be directed to the corresponding authors.
