# Supplementary material for: Tributyl phosphate as a type of environmental endocrine disruptor associated with liver fibrosis: insights from NHANES and in vitro validation
Source: Front Toxicol. 2025 Jul 1;7:1623830. doi: 10.3389/ftox.2025.1623830 (PMC12259707; doi:10.3389/ftox.2025.1623830)
Supplement: Supplementary file 1 [file DataSheet1.pdf]

Supplementary Material

Supplementary Table 1 The information of EEDs and their metabolites

EEDs and their liver toxicity	Metabolites	Detection rate
**Flame Retardants**		
Triphenyl phosphate(TPP)	Diphenyl phosphate (DPhP)	97%
Tris(1,3-dichloro-2-propyl) phosphate(TDCPP)	Bis(1,3-dichloro-2-propyl) phosphate (BDCPP)	97.3%
Tris(2-chloroethyl) phosphate(TCEP)	Bis-2-chloroethyl phosphate (BCEtP)	78.9%
Tributyl phosphate(TBP)	Dibutyl phosphate（DBuP）	52.9%
**Metal**		
Lead(Pb)		99.9%
Cadmium(Cd)		81.4%
Mercury(Hg)		73,5%
Selenium(Se)		100%
Manganese(Mn)		100%
**Organophosphate Insecticides**		
Dialkylphosphate	Dimethylphosphate（DMP）	96.8%
Chlorpyrifos	Diethylphosphate（DEP）	93.5%
	Dimethylthiophosphate（DMTP）	90.7%
Triazophos	Diethylthiophosphate（DETP）	58.8%
	Dimethyldithiophosphate（DMDP）	52.6%
**Perfluoroalkyl and Polyfluoroalkyl**		
Perfluorooctane sulfonamide（PFDeA）		88.6%
Perfluorohexane sulfonate（PFHxS）		99.3%
2-(N-Methyl-perfluorooctane sulfonamido) acetate （Me-PFOSA-AcOH）		59.0%
Perfluorononanoate		92.5%

（PFNA）		
Perfluoroundecanoic acid（PFUA）		66.0%
n-perfluorooctanoic acid（nPFOA）		99.6%
n-perfluorooctane sulfonic acid （nPFOS）		99.7%
Perfluoromethylheptane sulfonic acid isomers（SmPFOS）		99.2%
**Phthalates and Plasticizers**		
Di-isodecyl phthalate (DDP)	Monocarboxy-isononyl phthalate (MCNP)	96.2%
Di-isononyl phthalate (DNP)	Monocarboxyisooctyl phthalate (MCOP)	99.4%
Di(2-ethylhexyl) phthalate (DEHP)	Mono(2-ethyl-5-carboxypentyl) phthalate (MECPP)	99.8%
Di-n-butyl phthalate (DBP)	Mono-n-butyl phthalate (MBP)	99.3%
Di-n-butyl phthalate (DBP)	Mono (3-carboxypropyl) phthalate (MCPP)	83.1%
Di-ethyl phthalate (DEP)	Mono-ethyl phthalate (MEP)	99.5%
Di(2-ethylhexyl) phthalate (DEHP)	Mono(2-ethyl-5-hydroxyhexyl) phthalate (MEHHP)	99.0%
Di(2-ethylhexyl) terephthalate (DEHTP)	Mono(2-ethyl-5-hydroxyhexyl) terephthalate (MEHHTP)	96.6%
1,2-Cyclohexane dicarboxylic acid, diisononyl ester (DINCH)	Cyclohexane-1,2-dicarboxylic acid mono(hydroxy-isononyl) ester (MHINCH)	66.8%
Di(2-ethylhexyl) phthalate (DEHP)	Mono(2-ethylhexyl) phthalate (MEHP)	56.2%
Di-isobutyl phthalate (DiBP)	Mono-isobutyl phthalate (MiBP)	97.8%
Di(2-ethylhexyl) phthalate (DEHP)	Mono(2-ethyl-5-oxohexyl) phthalate (MEOHP)	99.2%
Di-isononyl phthalate (DNP)	Mono-oxo-isononyl phthalate (MONP)	80.6%
Benzylbutyl phthalate (BzBP)	Monobenzyl phthalate (MBzP)	96.2%
Di(2-ethylhexyl) terephthalate (DEHTP)	Mono(2-ethyl-5-carboxypentyl) terephthalate (MECPTP)	99.9%
1,2-Cyclohexane dicarboxylic acid, diisononyl ester (DINCH)	Cyclohexane-1,2-dicarboxylic acid mono(carboxyoctyl) ester (MCOCHP)	44.4%
Di-n-butyl phthalate (DBP)	Mono-3-hydroxy-butyl phthalate (MHBP)	97.9%
Di-isobutyl phthalate (DiBP)	Mono-2-methyl-2-hydroxypropyl phthalate(MHiBP)	95.1%

Supplementary Table 2 EED detection methods

EEDs	Methods
Flame Retardants	Solid phase extraction (SPE) coupled with isotope dilution ultrahigh performance liquid chromatography (UHPLC)-tandem mass
Metal	Mass spectrometry
Organophosphate Insecticides	Solid phase extraction (SPE) coupled with isotope dilution ultrahigh performance liquid chromatography (UHPLC)-tandem mass spectrometry
Perfluoroalkyl and Polyfluoroalkyl	Online solid phase extraction coupled to high performance liquid chromatography-turboionspray ionization-tandem mass spectrometry (online SPE-HPLC-TIS-MS/MS)
Phthalates and Plasticizers	High Performance Liquid Chromatography (HPLC)

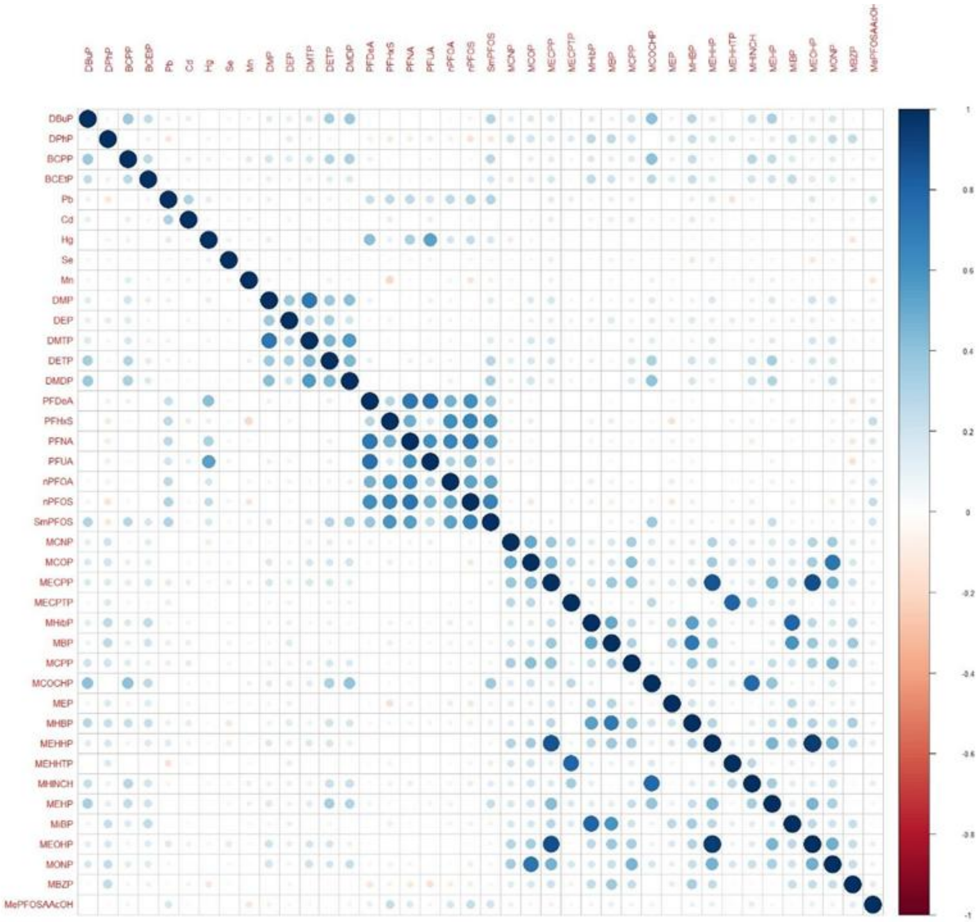

Supplementary Figure 1 The correlation between all variables of EEDs

Supplementary Table3 The correlation between variables which rspearman>0.8

	**MECPP**	**MEHHP**
**MEHHP**	0.85548326288*	
**MEOHP**	0.872386726*	0.948276101*

** P<0.05*

Supplementary Table4 The numbers of missing value of EEDs

EEDs	Numbers of missing value	EEDs	Numbers of missing value
DBuP	100	PFUA	98
BDCPP	97	nPFOA	105
DPhP	105	nPFOS	103
BCEtP	94	SmPFOS	131
Pb	57	MCNP	119
Cd	99	MECPP	83
Hg	120	MECPTP	149
Se	34	MHiBP	73
Mn	35	MCPP	94
DMP	109	MCOCHP	92
DEP	98	MEP	147
DMTP	125	MEHHP	83
DETP	122	MEHHTP	148
DMDP	137	MHINCH	118
PFDeA	102	MEHP	72
PFHxS	107	MiBP	87
Me-PFOSA-AcOH	101	MEOHP	81

PFNA	85	MBzP	111
MCNP	96	MONP	73
MHBP	105		

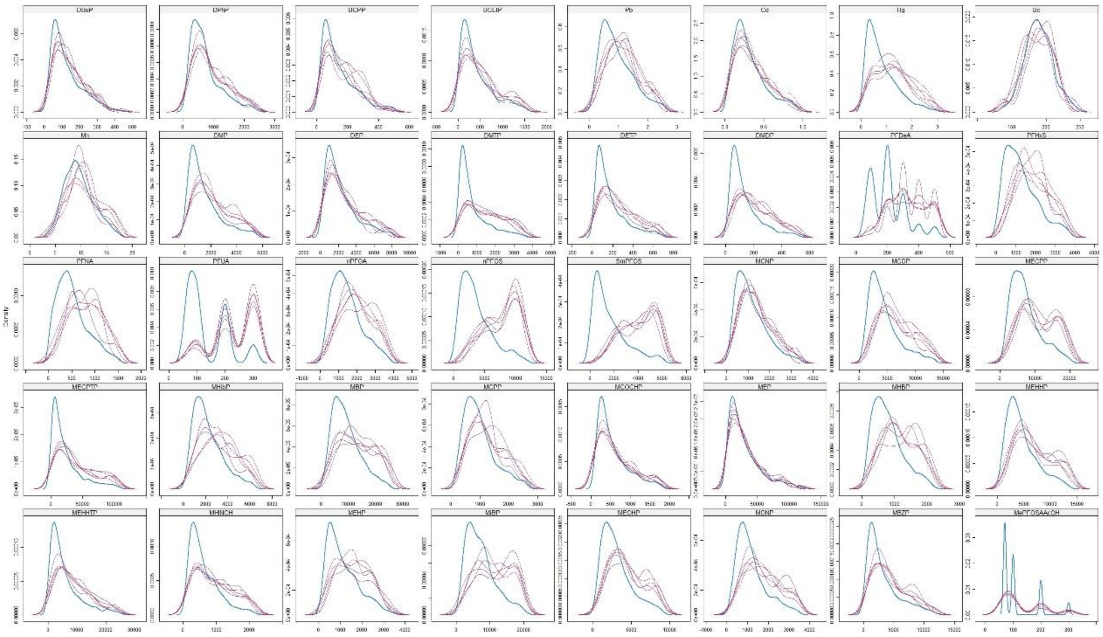

Supplementary Figure 2 The density chart of variables

The distribution of EEDs and their metabolites. The blue line indicating raw data and the red line indicating imputed data.

Supplementary Table 5 Primer sequence of Q-PCR

Gene	Primer	Sequence(5’-3’)
MMP2	Forward	GTGACGGCTTCCTCTGGTGTTC
	Reverse	CAGGGCTGTCCATCTCCATTGC
MMP9	Forward	CTCCTGGTGCTCCTGGCTCTAG
	Reverse	GCTGTGTGTCCGTGAGGTTGG
GAPDH	Forward	GTCGGTGTGAACGGATTTG
	Reverse	TCCCATTCTCAGCCTTGAC

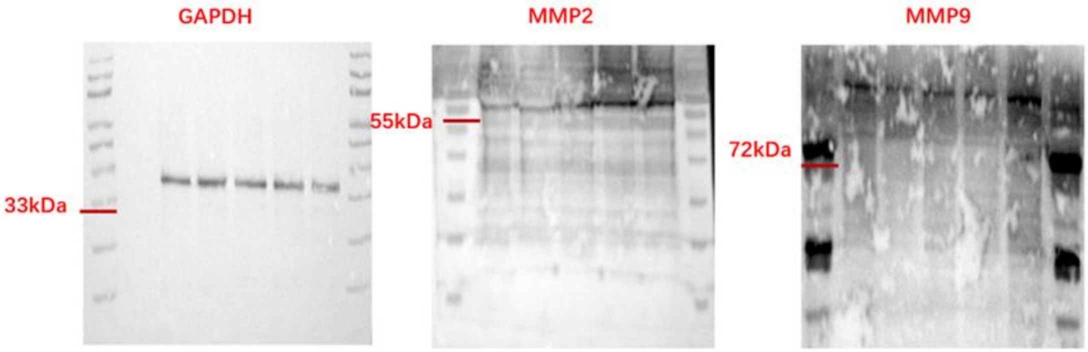

Supply Figure 3 Negative and positive controls for antibodies

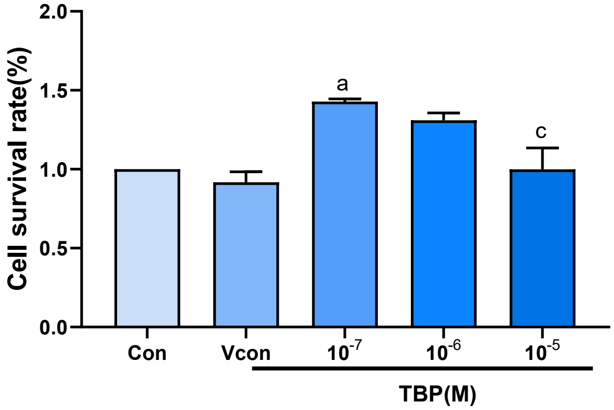

Supplementary Figure 4 Survival rate of BRL-3A cells

[a]Comparison with Con($P<0.05$), [b]Comparison with Vcon($P<0.05$) , [c]Comparison with 10^{-7}($P<0.05$), [d]Comparison with 10^{-6}($P<0.05$) . Values are means±SD (n≥3).

Supply Table 6 The levels of EEDs in human urine or blood with or without liver fibrosis

EEDs	Total	Fibrosis	Non-Fibrosis	p^a
**Flame Retardants-Urine (ng/g Cr)**				
DBuP	100[60.95, 172.76]	79 [53.19, 158.69]	100 [62.46, 176.75]	0.024
DPhP	620 [391.38, 974.19]	610 [411.89, 1186.03]	620 [389.11, 961.42]	0.531
BCPP	110 [57.95, 203.12]	120[57.95, 217.93]	110 [57.95, 202.00]	0.746
BCEtP	290 [141.40, 589.47]	290 [106.03, 579.52]	290 [143.33, 590.55]	0.437
**Metal-Blood (ug/L)**				
Pb	0.84 [0.52, 1.30]	0.86 [0.51, 1.30]	0.84 [0.53, 1.30]	0.882
Cd	0.27[0.17, 0.43]	0.24 [0.16, 0.37]	0.27 [0.17, 0.43]	0.222
Hg	0.67 [0.36, 1.21]	0.71 [0.38, 1.15]	0.66 [0.36, 1.21]	0.748
Se	190 [175.92, 203.59]	190 [176.08, 206.70]	190 [175.94, 203.10]	0.521
Mn	9.2 [7.51, 11.40]	9.0 [7.50, 10.97]	9.2 [7.51, 11.53]	0.570
**Organophosphate Insecticides-Urine (ng/g Cr)**				
DMP	1200 [630.47, 2149.70]	1200 [557.12, 2191.58]	1200 [642.55, 2142.86]	0.545
DEP	1800 [947.76, 3220.59]	1700 [907.11, 3099.48]	1800 [950.48, 3232.50]	0.386
DMTP	510 [232.08, 1271.19]	440 [180.30, 983.42]	520 [235.67, 1290.09]	0.109
DETP	140 [69.14, 252.76]	97 [56.11, 232.61]	140 [70.21, 259.99]	0.05
DMDP	110 [60.95, 197.96]	83 [53.16, 153.56]	110 [62.31, 204.11]	0.009
**Perfluoroalkyl and Polyfluoroalkyl-Urine(ng/L)**				
PFDeA	200 [100.00, 300.00]	200 [100.00, 250.00]	200 [100.00, 300.00]	0.031
PFHxS	1100 [600.00, 1800.00]	1200 [600.00, 1700.00]	1100 [600.00, 1900.00]	0.743
PFNA	500 [300.00, 700.00]	500 [300.00, 600.00]	500 [300.00, 700.00]	0.8
PFUA	100 [70.00, 200.00]	100 [70.00, 200.00]	100 [70.00, 200.00]	0.495
nPFOA	1300 [900.00, 2000.00]	1300 [900.00, 1800.00]	1400 [900.00, 2,000.00]	0.566
nPFOS	3300 [1900.00, 5700.00]	3200 [1550.00, 5150.00]	3300 [1900.00, 5700.00]	0.227
SmPFOS	1400 [636.36, 2650.60]	1300 [632.73, 2595.00]	1400 [636.85, 2682.93]	0.785
**Phthalates and Plasticizers Metabolites-Urine (ng/g Cr)**				
MCNP	1000 [641.03, 1559.63]	1100 [655.31, 1795.63]	1100 [640.68, 1538.46]	0.103

MCOP	3800 [2476.19, 6000.00]	4500 [2784.24, 6240.32]	3800 [2472.06, 5926.43]	0.141
MECPP	6600 [4318.18, 9864.86]	7900 [4967.26, 10400.64]	6500 [4249.27, 9754.57]	0.033
MECPTP	16000 [6716.42, 35428.57]	18000 [6888.59, 34784.05]	16000 [6662.33, 35461.24]	0.553
MHibP	1900 [1219.51, 3068.18]	1700 [1116.37, 2562.85]	2000 [1233.80, 3134.19]	0.012
MBP	8300 [5241.38, 12500.00]	8.000 [5404.17, 11089.93]	8400 [5195.56, 12665.79]	0.599
MCPP	900 [598.29, 1300.81]	960[645.76, 1434.11]	900 [597.94, 1291.48]	0.196
MCOCHP	430 [263.16, 769.23]	380 [249.14, 625.00]	430 [263.16, 769.23]	0.257
MEP	22000 [11188.81, 41545.45]	20000[11954.72, 39052.32]	22000 [11166.77, 41518.80]	0.662
MHBP	730 [467.29, 1097.56]	630 [414.84, 931.21]	740 [470.59, 1120.00]	0.036
MEHHP	400 [2619.05, 6415.09]	3900 [2738.78, 6691.00]	4000 [2594.65, 6401.91]	0.722
MEHHTP	4300[2187.50, 8415.09]	4700 [2755.89, 8980.82]	4300 [2106.16, 8375.00]	0.104
MHINCH	520[307.69, 909.09]	450 [275.46, 809.49]	540 [314.61, 916.03]	0.084
MEHP	970[566.80, 1628.57]	950 [500.02, 1492.49]	980 [570.00, 1630.83]	0.342
MiBP	6800 [4061.30, 10078.74]	6700 [3750.89, 9659.09]	6.800 [4096.37, 10086.21]	0.735
MEOHP	2700[1639.34, 4210.53]	2800 [1745.30, 4261.65]	2700 [1631.80, 4206.91]	0.636
MONP	1100 [681.82, 1617.65]	1200 [845.08, 1686.05]	1.000 [674.16, 1612.36]	0.025
MBZP	2400 [1301.37, 4497.04]	2800 [1526.81, 4697.86]	2400[1269.84, 4485.54]	0.234
MePFOSAAcOH	100[70.00, 200.00]	100 [70.00, 100.00]	100 [70.00, 200.00]	0.478

Data are presented as the median (Inter Quartile Range).

[a] Rank sum test was performed for continuous variables.
